# Genetic Cross-Talk between Oral Squamous Cell Carcinoma and Type 2 Diabetes: The Potential Role of Immunity

**DOI:** 10.1155/2022/6389906

**Published:** 2022-05-19

**Authors:** Yunjian Fan, Jie Zhang, Jiayu Shi, Lin Chen, Jiazhen Long, Shuqi Zhang, Shuguang Liu

**Affiliations:** Stomatology Hospital, Southern Medical University, Guangzhou 510280, China

## Abstract

**Background:**

This bioinformatics study was aimed at evaluating type 2 diabetes (T2D) and oral squamous cell carcinoma (OSCC) with regard to related immune cells and prognosis.

**Methods:**

We downloaded the data on OSCC from TCGA and for T2D from GEO database. Differentially expressed genes were analyzed, i.e., for OSCC genes with *p* value < 0.01, |log2(FC)| > 0; and for T2D, genes with *p* value < 0.05, |log2(FC)| > 0. The intersected genes between OSCC and T2D were cross-talk genes. The expression values of immune-related genes in case samples in OSCC and T2D were assessed and underwent multivariate and univariate analysis (Cox-PH model). The intersection between the immune genes and cross-talk genes was taken and further analyzed by recursive feature elimination (RFE), survival analysis, and ROC analysis.

**Results:**

1008 cross-talk genes were acquired, including 28 common upregulated, 440 common downregulated, and 540 differently regulated DEGs. We extracted the gene expression value of 782 immune-related genes, of which seven increased immune cells were obtained. From the results, plasmacytoid dendritic cells and effector memory CD8 T cells were highly negatively correlated in both OSCC and T2D. After estimating a low- and high-risk model for survival, we found that activated dendritic cell was significantly different between high and low groups (*p* = 0.0095), followed by plasmacytoid dendritic cell. We integrated DE_Immune genes set 1 and DE_Immune genes set 2 and eight key immune-related cross-talk genes (C1QC, ABCD1, NOS2, PDIA4, IL1RN, ALOX15, CSE1L, and PSMC4) were evaluated. After ROC analysis, we obtained that ABCD1, C1QC, CSE1L, and PSMC4 had higher classification and prediction effects on OSCC and T2D.

**Conclusion:**

This study revealed a close relationship between T2D and OSCC. Thereby, plasmacytoid dendritic cell and activated dendritic cell-related genes were associated with the survival of T2D-related OSCC, while ABCD1, C1QC, CSE1L, and PSMC4 were the most important immune-related cross-talk genes.

## 1. Background

Diabetes mellitus, especially type 2 (T2D), is an enormous global health issue, whereby over 90% out of the 415 million individuals suffering from diabetes worldwide have a frank T2D [1]. In course of T2D, the sensitivity of the insulin receptors, regulating the glucose inclusion into cells, is decreased, resulting in hyperglycemia as main symptom [2]. This is associated with lifestyle of the patients, including nutritional behavior, physical education, increased body weight, or smoking as risk factors [2]. Therapy and management of T2D developed into a large aim of current research and promising approaches are repeatedly introduced [3]. However, diabetes-related diseases are common and of high clinical relevance; those include microvascular and macrovascular complications, alongside with oral diseases like increased periodontal inflammation and oral mucous diseases [1, 4].

Another potentially diabetes-related problem is the occurrence and prognosis of oral cancer, especially oral squamous cell carcinoma (OSCC) [5]. This common form of head and neck cancer is often related with high morbidity and, especially in advanced stages, poor prognosis [6]. Thereby, potential factors affecting the risk of development of OSCC, as well as prognostic parameters/models for OSCC outcome, are of high research interest [6]. Different mechanisms between T2D and OSCC have been mentioned in literature, including promoted proliferation, metastasis formation, and suppression of apoptosis of OSCC in case of diabetes [5]. Although it has been reported that T2D alone appears not to affect OSCC survival, the evidence is limited due to contradictory results [7]. To understand the interplay between T2D and OSCC, further research is required, especially with regard to cellular and molecular mechanisms [8]. Especially, the role of inflammation and thus inflammatory tumor environment appears of interest, as this is an interesting and promising field of research [9].

Recently, bioinformatics was repeatedly used to evaluate both, OSCC prognosis and its cross-talk with other diseases and conditions, like e.g., periodontitis [10, 11]. This kind of research allows a comprehensive and deep insight in the topic and helps to identify potential biomarkers, which can be a basis for future research in the field. Against this background, this bioinformatics study followed the objective to reveal the cross-talk between T2D and OSCC. For this, the focus was on immune cells or immune-related cells, to identify the role of inflammation and immunity in the interplay between the two diseases. Finally, models for the prediction of survival were estimated.

## 2. Materials and Methods

### 2.1. Datasets

We downloaded the RNA-seq dataset of Head and Neck Squamous Cell Carcinoma (HNSC) and corresponding clinical data from TCGA (https://portal.gdc.cancer.gov/). Based on the anatomic neoplasm subdivision results in the clinical information of HNSC, we selected samples related to the buccal mucosa, alveolar ridge, floor of the mouth, hard palate, oral cavity, and tongue, which were classified as oral squamous cell carcinoma (OSCC). Gene expression profiling data for Type 2 Diabetes (T2D) in humans was downloaded from the GEO database (http://www.ncbi.nlm.nih.gov/). Finally, three datasets of T2D were obtained: GSE23561, GSE26168, and GSE184050. We used the data of GSE184050 to analyze the cross-talk genes, while GSE23561 and GSE26168 acted as the validation sets (Table 1). Immune genes and immune cells were obtained from the literature [PMID: 28052254]. Finally, 782 immune genes were obtained, which corresponded to 28 immune cell types.

### 2.2. Data Preprocessing

For the OSCC obtained from the TCGA database, we converted the ensemble ID to gene symbol based on the annotation file from GENCODE (https://www.gencodegenes.org/human/). When multiple ENSG ID were mapped on one gene, the mean expression value of ENSG ID in samples was taken as the gene expression value.

For the microarray dataset of T2D obtained from the GEO database, we first converted the probes into corresponding gene names based on the platform information. When multiple probes corresponded to the same gene, we selected the mean of the expression values of these probes in a certain sample as the expression value of the gene in this sample. Finally, if the number of samples under a gene with a zero value exceeded 50% of the total number of samples, we deleted the gene from the dataset.

### 2.3. Differentially Expressed Gene Analysis

The “edgeR” package of R project was applied to analyze the differentially expressed genes for OSCC and T2D. For the OSCC dataset, genes with *p* value < 0.01, |log2(FC)| > 0 were differentially expressed genes which were dysregulated between control samples versus case samples. For the T2D dataset, genes with *p* value < 0.05, |log2(FC)| > 0 were selected as differentially expressed genes.

### 2.4. Cross-Talk Genes

We extracted the intersection of DEGs of OSCC and T2D, which are cross-talk genes of OSCC and T2D. The expression values of cross-talk genes in OSCC and T2D were then extracted, and the “pheatmap” package of R project was used to show the expression levels of these genes in the samples. Then, the “clusterProfiler” package of R project was used to analyze the GO Biological processes and KEGG pathways of cross-talk genes. The functions of the *p* value < 0.05 were the significant functions.

### 2.5. Immune Cell Enrichment of OSCC and T2D

We extracted the expression values of immune-related genes in case samples in OSCC and T2D. To analyze the relationship between immune genes and immune cells, we performed ssGSEA analysis for the sample expression values of immune genes using the GSVA package of R. Sample enrichment scores for immune cells were obtained by ssGSEA analysis. We combined the immune cell enrichment scores of OSCC and T2D and obtained the immune cells with higher enrichment scores through hierarchical clustering.

### 2.6. Multivariate Analysis of Immune Cells (High) Based on Cox-PH Model

To investigate the relationship between immune cell scores and survival in OSCC, we obtained overall survival and survival events from clinical information of OSCC. A multivariate Cox proportional risk regression model (Cox-PH) was then built on immune cell scores, overall survival, and survival events using the “survival package” of R. Out of this model, we can obtain the Hazard ratio of each feature variable and the risk score of the sample. After the model is established, the ROC analysis of the model was first performed to assess its prediction effect. We performed the ROC analysis with “timeROC” package of R based on risk score, overall survival, and survival event. Meanwhile, we divided the samples into high-risk groups and low-risk groups based on the median risk score. The survival analysis of the two risk groups was carried out by using the “survival” package of R, and the survival analysis results were presented by the “survminer” package of R.

### 2.7. Survival Analysis for Immune Cells Based on the Univariate Cox-PH Model

Each immune cell in univariate Cox-PH model acted as a feature variable. We built a model for each feature variable and predicted the relationship between feature variable and survival through the risk score of the model. We analyzed each immune cell (high) using a univariate Cox-PH model to obtain a risk score and divided the samples into high-risk groups and low-risk groups for survival analysis based on the median risk score.

### 2.8. Immune-Related Cross-Talk Gene

The intersections between the immune genes and cross-talk genes were taken, and the intersected genes were the immune-related cross-talk genes. We performed the function enrichment including GO biological process and KEGG pathway with the “clusterProfiler” package of R project. Besides, we extracted the genes form the immune cells, which were highly related with survival and these genes were noted as *DE_Immune genes set 1*.

### 2.9. Gene Screening with Recursive Feature Elimination (RFE)

We extracted the gene expression value of immune-related cross-talk genes from OSCC and T2D and then performed the RFE for the case and control group of samples with the rfe method of “caret” of R project. With the RFE, we obtained the key immune-related cross-talk genes of OSCC and T2D, which were noted as *DE_Immune genes set 2.*

### 2.10. The Survival Analysis and ROC Analysis for the Immune-Related Cross-Talk Genes

We acquired the DE_Immune genes by integrating *DE_Immune genes set1* and *DE_Immune genes set2.* Then, we extracted the gene expression value of DE_Immune genes in case group of OSCC and built the Cox-PH model of univariate analysis to analyze the relationship between gene and survival. We extracted the expression values of DE_Immune genes in case and control sample of OSCC and T2D and performed ROC analysis to analyze the AUC of these genes.

## 3. Results

### 3.1. Differential Expression Analysis

**Figure 1:**
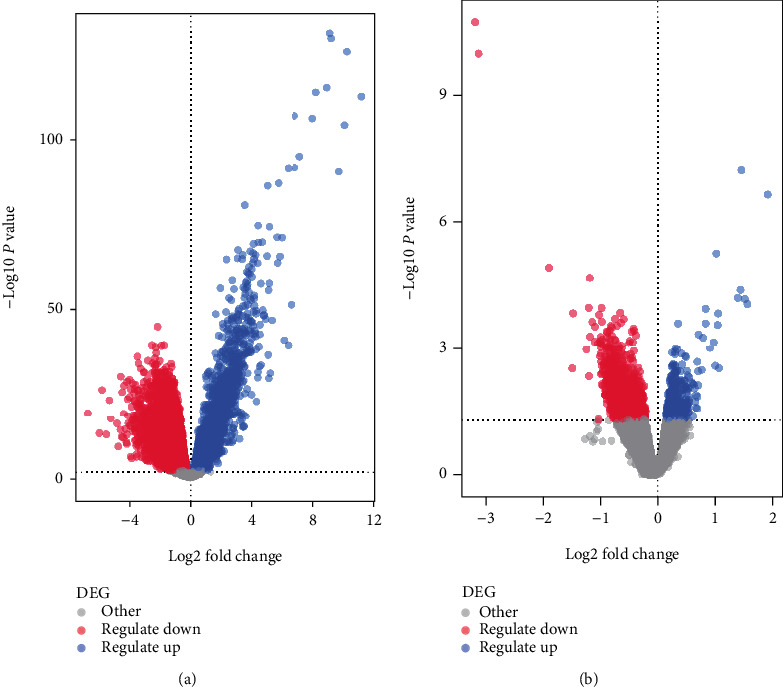
Volcano plot of DEGs in OSCC (a) and T2D (b).

For OSCC, we selected genes with *p* value < 0.01, |log2(FC)| > 0 as differentially expressed genes (DEG), where Log2(FC) > 0 is an upregulated and log2(FC) < 0 is a downregulated gene (Figure 1(a)). For the T2D dataset, the genes with *p* value < 0.05 and |log2(FC)| > 0 acted as DEGs, where Log2(FC) > 0 was an upregulated and log2(FC) < 0 was a downregulated gene (Figure 1(b)). The counts of DEG are shown in Table 2.

### 3.2. Cross-Talk Genes between OSCC and T2D

**Figure 2:**
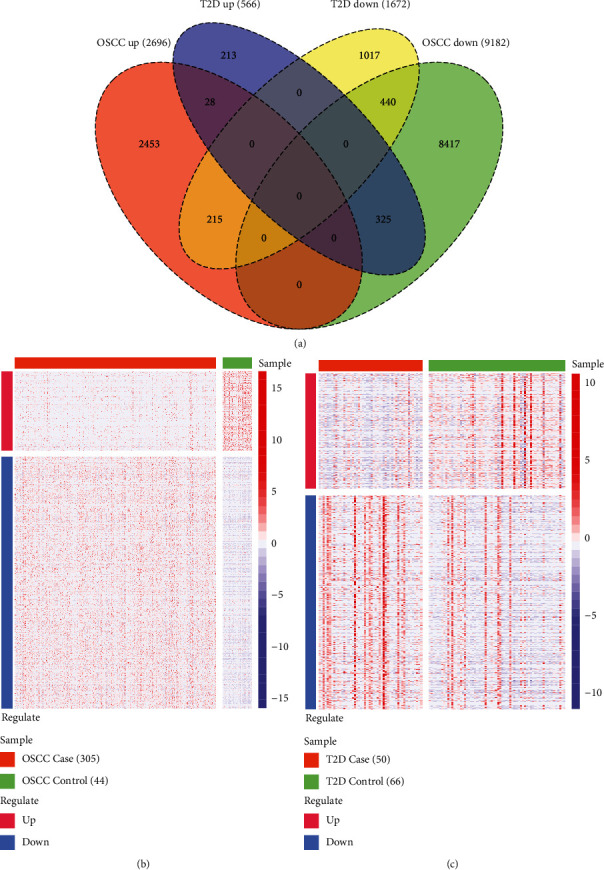
Cross-talk gene counts (a) and the expression level of cross-talk gene in OSCC (b) and T2D (c).

**Figure 3:**
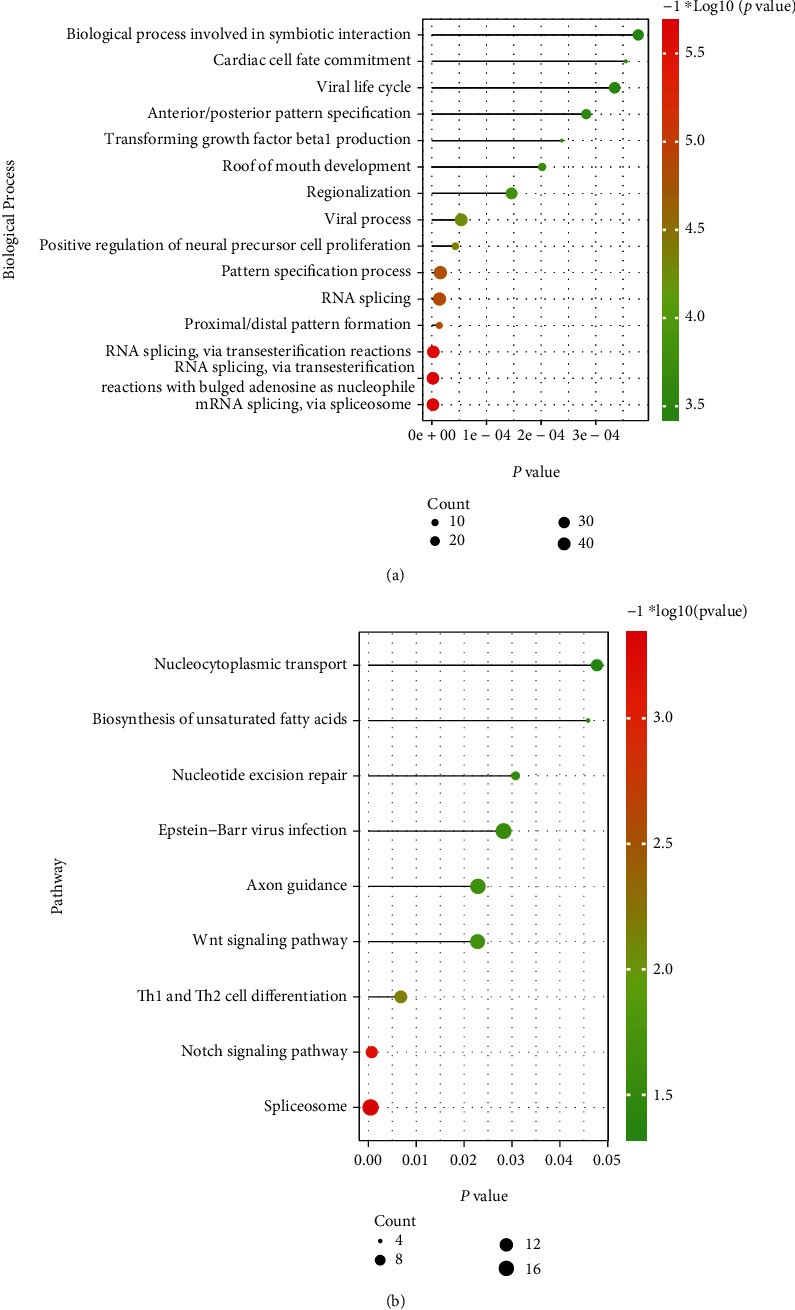
The function enrichment of cross-talk genes. (a) The significant enriched biological processes and (b) the significant enriched KEGG pathways.

We extracted the common DEGs between OSCC and T2D and 1008 cross-talk genes were acquired including 28 common upregulated DEGs, 440 common downregulated DEGs, and 540 differently regulated DEGs (Figure 2(a)). We extracted the gene expression value of cross-talk genes from OSCC and T2D and showed the gene expression level with heat map (Figures 2(b) and 2(c)). With the “clusterProfiler” package of R project, we analyzed the enriched functions of cross-talk genes (Figure 3). The results showed that cross-talk genes regulated the RNA splicing, pattern specification process, biological process involved in symbiotic interaction, and so on. Meanwhile, the cross-talk genes were involved in the Notch signaling pathway, Th1 and Th2 cell differentiation, Wnt signaling pathway, and so on.

### 3.3. The Enrichment of Immune Cells

**Figure 4:**
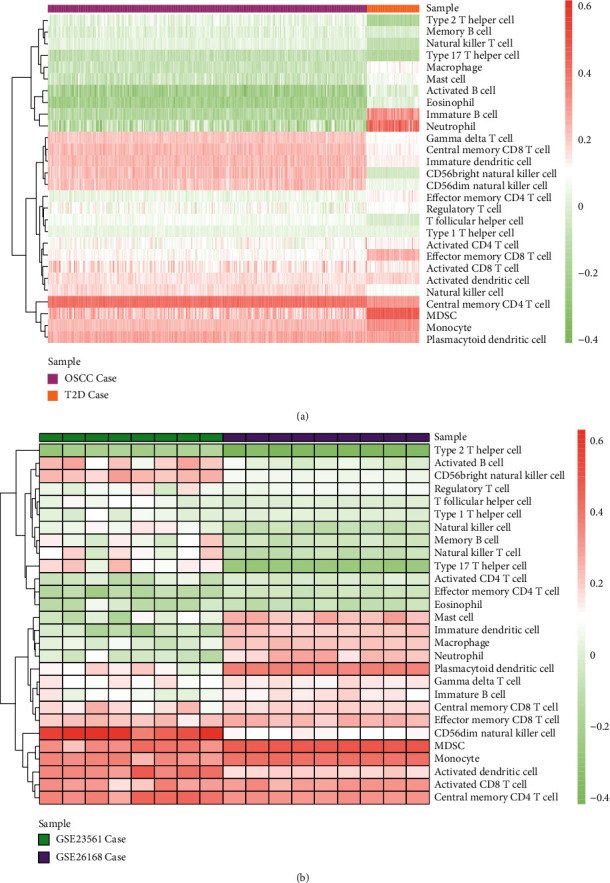
Immune cell enrichment analysis of OSCC and T2D. (a) The enrichment analysis for immune cells in OSCC and T2D and (b) the enrichment analysis for immune cells in other two datasets of T2D.

We extracted the gene expression value of 782 immune-related genes in the case group of OSCC and T2D and performed the enrichment of immune cells with ssGSEA. We integrated the immune cell enrichment scores of OSCC and T2D and then obtained the higher enrichment scores of immune cells by hierarchical clustering (Figure 4(a)). In addition, we performed the immune cell enrichment for T2D datasets GSE23561 and GSE26168 and selected the immune cells with higher enrichment score through hierarchical clustering (Figure 4(b)).

**Figure 5:**
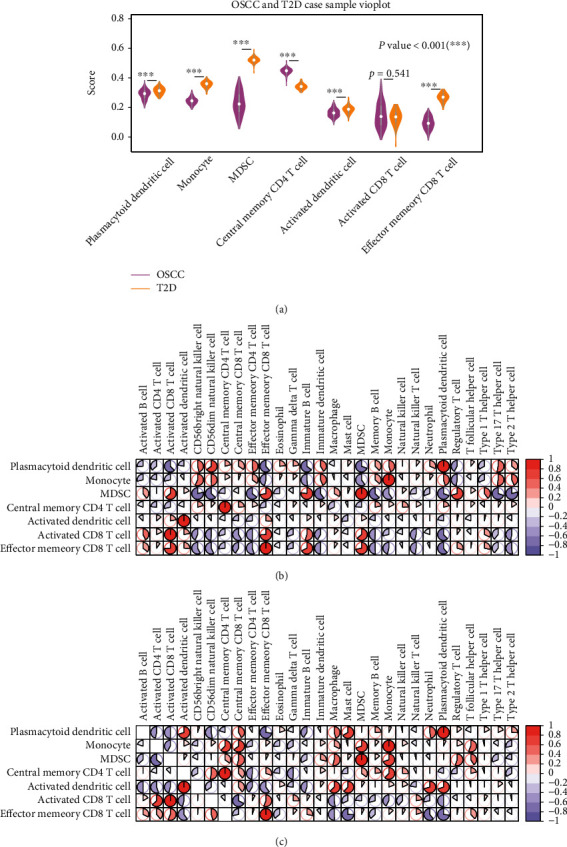
Seven high immune cells. (a) The enrichment and differences of seven high immune cells in OSCC and T2D. The horizontal axis represents immune cells, and the vertical axis represents the enrichment score. ^∗^Represents the difference of immune cells between in OSCC and T2D: ^∗^: *p* < = 0.05, ^∗∗^: *p* < = 0.01, ^∗∗∗^: *p* < = 0.001, ^∗∗∗∗^: *p* < = 0.0001. (b) Correlation of 7 high immune cells and other immune cells in OSCC. (c) Correlation of 7 high immune cells and other immune cells in T2D.

Seven increased immune cells were obtained, which were plasmacytoid dendritic cell, central memory CD8 T cell, MDSC, monocyte, activated dendritic cell, activated CD8 T cell, and central memory CD4 T cell. We used the violin diagram to show the distribution of increased immune cells and performed a Wilcoxon test on the sample fractions of OSCC and T2D (Figure 5(a)). The results showed that only activated CD8 T cells had no significant difference between OSCC and T2D. We analyzed the correlation between the seven increased and other immune cells (Figures 5(b) and 5(c)). From the results, plasmacytoid dendritic cells and effector memory CD8 T cells were highly negatively correlated in both OSCC and T2D.

### 3.4. Multivariate Cox-PH Analysis of Immune Cells

**Figure 6:**
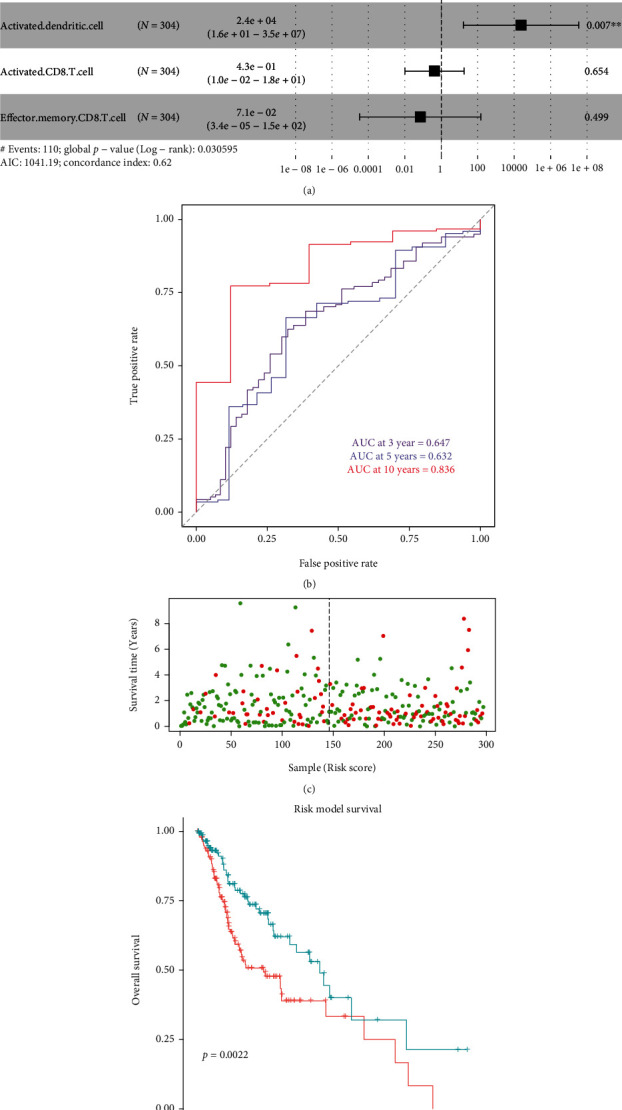
Multivariate Cox-PH analysis of high immune cell. (a) Risk forest plot of 7 immune cells. (b) Multivariate model assessment of the predictive effect of 3 years, 5 years, and 10 years. (c) The relationship between survival time and risk. Red points are dead samples and green points are surviving samples. The left side of the dotted line is the low-risk group sample, and the right side of the dotted line is the high-risk group sample. (d) Survival analysis of the high- and low-risk group.

We obtained 304 samples with survival time from OSCC and then used the enrichment scores of seven increased immune cells in these samples to build a multivariate Cox-PH model. After building the model, we checked the Hazard ratio of immune cells in the model through the forest plot (Figure 6(a)). The results showed that plasmacytoid dendritic cell and activated dendritic cell were more significant in the model. Among of the immune cells, plasmacytoid dendritic cell correlated with lower risk, while activated dendritic cell correlated with higher risk.

In order to evaluate the prediction effect of the model, we used the timeROC package to integrate Hazard ratio, overall survival, and survival event and then performed ROC analysis for 3 years, 5 years, and 10 years, respectively (Figure 6(b)). From Figure 6(b), it can be seen that the prediction effect of the model is better (83.6%) in the sample within 10 years. Therefore, we selected samples of overall survival within 10 years from OSCC, obtaining 297 samples, which were divided into high-risk group and low-risk group according to the median risk score (Figure 6(c)). Subsequently, we performed a survival analysis on the high-risk group and the low-risk group, and there was a significant difference between the high-risk group and the low-risk group (*p* = 0.0022), with a significantly lower survival rate of the high-risk group than of the low-risk group (Figure 6(d)).

### 3.5. Univariate Cox-PH Analysis for High Immune Cells

**Figure 7:**
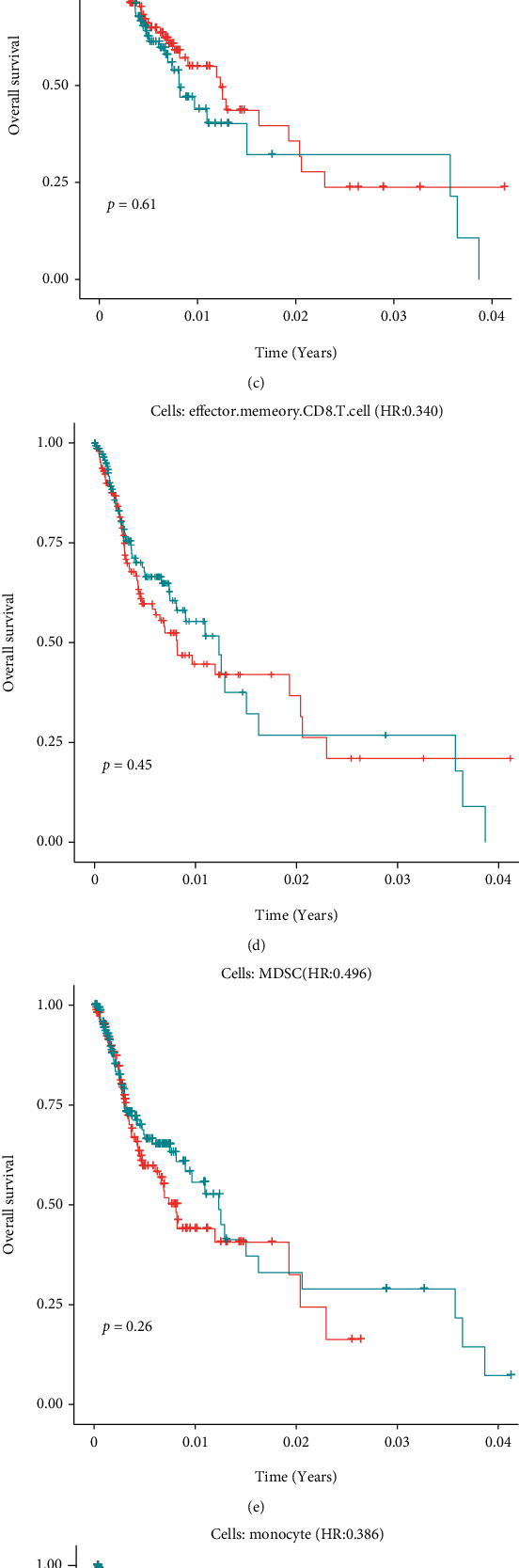
The result of univariate Cox-PH analysis for 7 high immune cells.

We treated each high immune cell as a characteristic variable to build a Cox-PH model and then divided the samples into high-risk and low-risk groups, according to the median risk score for survival analysis (Figure 7). In Figure 7, we found that activated dendritic cell was significantly different between high and low groups (*p* = 0.0095), followed by plasmacytoid dendritic cell, which was consistent with our multivariate analysis results. The results showed that activated dendritic cells and plasmacytoid dendritic cells were associated with survival.

### 3.6. Immune-Related Cross-Talk Gene

**Figure 8:**
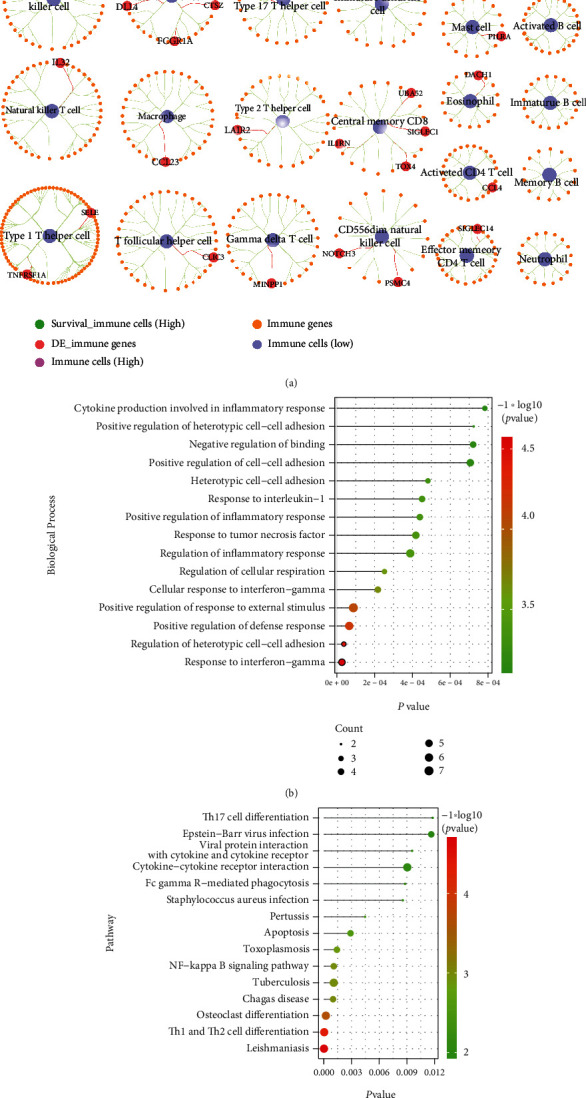
Results of immune-related cross-talk gene analysis. (a) 7 high immune cells and gene relationships; (b) GO biological process with significant enrichment of immune-related cross-talk genes; and (c) KEGG pathway with significant enrichment of immune-related cross-talk genes.

We took the intersection of 782 immune genes and 1008 cross-talk genes and obtained 50 immune-related cross-talk genes. We used Cytoscape to examine the relationship between 50 immune-related cross-talk genes and immune cells (Figure 8(a)). From Figure 8(a), we obtained plasmacytoid dendritic cell and activated dendritic cell-related genes (C1QC, ABCD1, NOS2, and PDIA4), which were marked as DE_Immune genes set 1. Next, we performed GO Biological process and KEGG pathway analysis on these 50 immune-related genes, selected *p* value < 0.05 as a significant function, and used a bubble chart to display these significant pathways (Figures 8(b) and 8(c)). These 50 immune-related cross-talk genes were mainly involved in biological processes such as response to interferon-gamma, regulation of heterotypic cell-cell adhesion, and positive regulation of defense response (Figure 8(b)). At the same time, these 50 immune-related cross-talk genes regulated Th1 and Th2 cell differentiation, NF-kappa B signaling pathway, TNF signaling pathway, pathways of neurodegeneration—multiple diseases, and other pathways (Figure 8(c)).

### 3.7. Further Screening of Immune-Related Cross-Talk Genes

**Figure 9:**
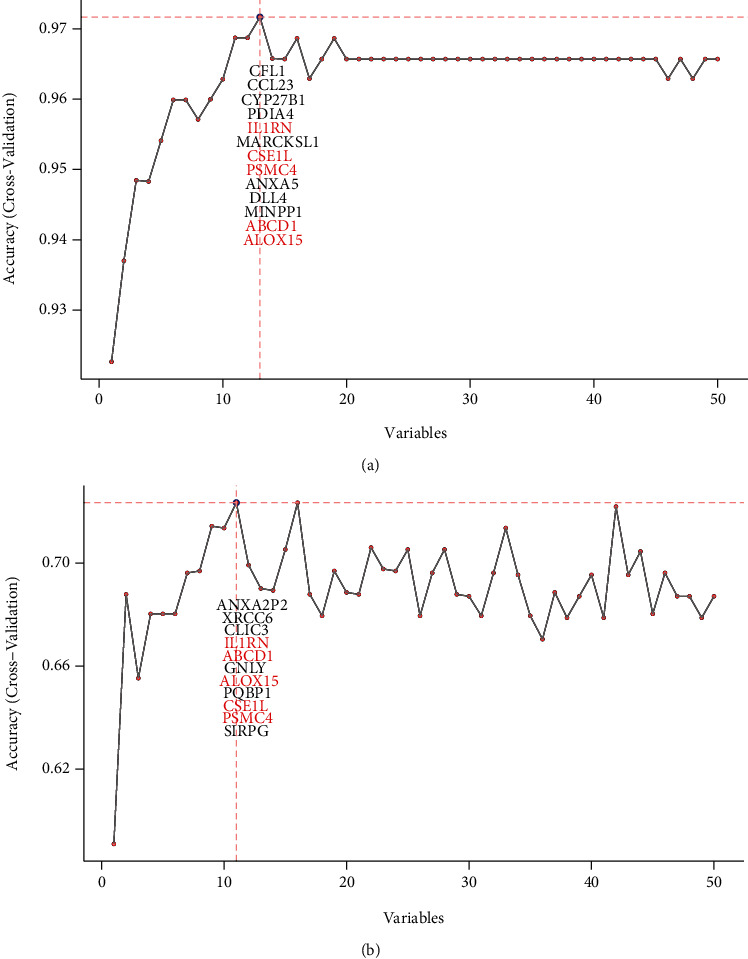
RFE signature screening of immune-related cross-talk genes in OSCC (a) and T2D (b). The abscissa represents the number of variables, and the ordinate represents the accuracy of feature selection.

We extracted the expression values of 50 immune-related cross-talk genes in OSCC and T2D and then performed feature screening using the RFE algorithm (Figure 9). Through analysis, 13 immune-related cross-talk genes were obtained from OSCC (Figure 9(a)) and 11 immune-related cross-talk genes were obtained from T2D (Figure 9(b)). There were five common genes (IL1RN, ABCD1, ALOX15, CSE1L, and PSMC4) in OSCC and T2D, which we marked as DE_Immune genes set 2.

### 3.8. Immune-Related Cross-Talk Genes for Survival Analysis and ROC Analysis

**Figure 10:**
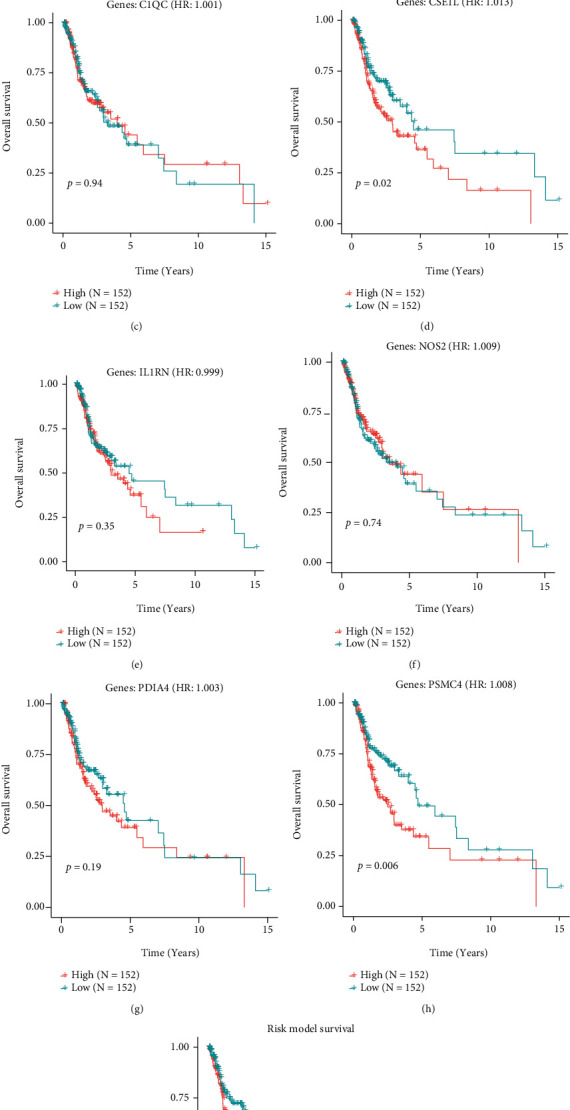
Survival analysis results of 8 key immune-related cross-talk genes.

We integrated *DE_Immune genes set 1* and *DE_Immune genes set 2* and eight key immune-related cross-talk genes (C1QC, ABCD1, NOS2, PDIA4, IL1RN, ALOX15, CSE1L and, PSMC4). Then, a univariate Cox-PH model was constructed for these eight genes to see the relationship between these genes and survival (Figures 10(a)–10(h)). We obtained that CSE1L and PSMC4 had significant differences in survival in different risk groups. The differences of other genes in different risk groups were not obvious. It is possible that they interact with other genes to affect survival. Therefore, we combined these eight genes to establish a multivariate Cox-PH model for survival analysis, and the results showed that these eight genes had significant differences in survival (Figure 10(i)).

**Figure 11:**
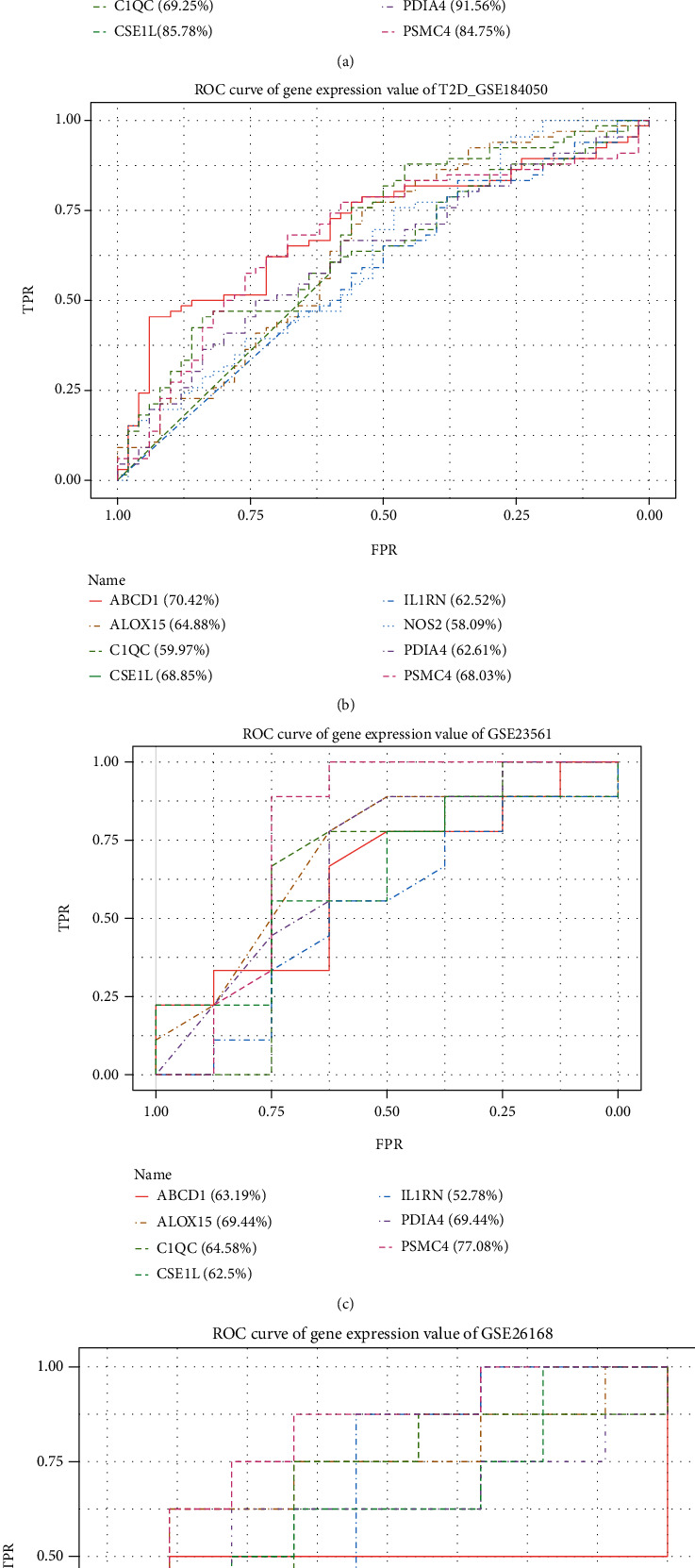
ROC analysis of 8 key immune-related cross-talk genes in OSCC, T2D, and validation datasets. GSE23561 and GSE26188 are validation datasets for T2D.

We extracted the expression values of these eight genes in case and control groups of OSCC and T2D and extracted their expression values in the validation sets GSE23561 and GSE26168. ROC analysis was performed on the four datasets to see the predictive effect of these gene expression values (Figure 11). After ROC analysis, we obtained that ABCD1, C1QC, CSE1L, and PSMC4 had higher classification and prediction effects on OSCC and T2D when the sample size was large. The four genes, ABCD1, C1QC, CSE1L, and PSMC4, are immune-related genes and are differentially expressed in T2D and OSCC, playing a role as a bridge between the two diseases.

## 4. Discussion

In this bioinformatics study, we identified a genetic cross-talk between T2D and OSCC, confirming their close relationship. Regarding prognosis, a high-risk and a low-risk model for survival was estimated; thereby, plasmacytoid dendritic cell- and activated dendritic cell-related genes were associated with the survival of T2D-related OSCC. Four genes, i.e., ABCD1, C1QC, CSE1L, and PSMC4, were the most important immune-related cross-talk genes between T2D and OSCC.

Based on recent literature, a genetic cross-talk between T2D and OSCC was expectable and thus the aim of this current research project [5]. It is repeatedly documented that T2D is a risk factor for the development of OSCC [12]. Moreover, T2D is related with metastasis formation and prognosis of OSCC [5]. Additionally, the diabetes-related therapy, e.g., metformin intake has an effect on the relationship between OSCC and T2D [13, 14]. Thereby, the causality of the relationship or, in other words, whether T2D would be an independent predictor for OSCC formation and outcome can be discussed controversially [7]. An indirect link would also be plausible; on the one hand, a shared risk complex, i.e., nutrition, obesity, and smoking would also predict T2D as well as OSCC [15–19]. On the other hand, oral inflammations, especially periodontitis and mucous diseases, are related to T2D [4, 20]. Those disease, however, are also closely related to OSCC [21, 22]. Considering the close cross-talk between OSCC and periodontitis, as revealed by another bioinformatics study [10], a complex interaction between the different chronic diseases appears probable. This makes a clear statement on the potential causality vs. coincidence very difficult.

Against this background, however, the role of inflammation between T2D and OSCC is worth to be recognized. As revealed in our study, dendritic cells were found to be of highest relevance in this context. Thereby, lower expression of the immune genes for activated and plasmocytoid dendritic cells was associated with better survival in the patients. Dendritic cells have a crucial function in induction of the protective adaptive immunity, making it a focus of cancer immunology and therapy [23]. Dendritic cells are regulators of the adaptive immune response and represent a heterogeneous population of leukocytes [24]. Those cells can be divided into conventional and plasmocytoid dendritic cells, having distinct functions in immunology [25]. Different types of dendritic cells have also been already examined in OSCC [26–28]. A recent study based on flow cytometry found that an increase in tumor-infiltrating plasmocytoid cells promotes OSCC proliferation, primarily by TNF-*α*/NF-*κ*B/CXCR-4 pathway [27]. Another study directly showed an increase of those cells to be related to poor OSCC prognosis [29]. Therefore, an association of the reduced expression of dendritic cell genes with better survival in the current study appears plausible. Dendritic cells are also related with T2D. Especially in obese individuals, dendritic cells are involved in the development of an adipose tissue inflammation, leading to insulin resistance [30]. Another study in mice found that dendritic cells drive mechanisms to increase the insulin reserve in response to obesity-induced insulin resistance [31]. Accordingly, an increased activation of dendritic cells, especially in adipose individuals with T2D, might affect the relationship between T2D and OSCC and its prognosis, respectively.

Furthermore, the four immune-related genes ABCD1, C1QC, CSE1L, and PSMC4 require discussion. ABCD1 is one of three members of the D subfamily of the ATP-binding cassette (ABC) transporters, which act as tetramers in peroxisome metabolism and thus in cell signaling and control [32]. While there is no research for this gene in oral cancer, some findings in literature indicate a role of ABCD transporters in cancer in general [33]. On the other side, a whole exome sequencing analysis revealed ABCD1, amongst other genes, as potentially relevant in context of T2D. However, the low body of evidence does not allow further hypotheses on the role of ABCD1 in the interplay between T2D and OSCC. C1QC, as important gene for the complement system and thus innate immune response, has been found to be a part of tumor microenvironment of different cancer entities [34, 35]. In esophageal squamous cell carcinoma, C1QC was potentially related with prognosis in a bioinformatics analysis [36]. Therefore, its relation with OSCC development and prognosis appears reasonable. Two hints for the potential role of C1QC in T2D are also available. One bioinformatics study found C1QC to be a potential hub gene, which is associated with immune cell infiltration related with the progression of obesity-related diabetes or insulin resistance, respectively [37]. This is similar as for dendritic cells, which was explained above. Considering that dendritic cells produce C1Q as initial molecule for the complement system [38], this interlink seems relevant in context of the current study. Moreover, the complement system-related genes were found to play a role in diabetic nephropathy [39]. Altogether, a potential role of complement system in the interplay between T2D and OSCC appears possible, but requires further data.

CSE1L (human chromosomal segregation 1-like), which is an effector of apoptosis, invasiveness, and migration of cancer cells, was already found to be related with oral cancer [40]. This has been shown to be regulated by MITF, which is upregulated, resulting in activation of the Akt/mTOR pathway [40]. No data regarding CSE1L and diabetes are available, making its role in T2D-related OSCC questionable. Similarly, PSMC4, which is a member of the ATPase gene (PSMC) family and thus involved in protein degradation, has been reported to be related with cancer development and prognosis [41, 42]. However, no literature supports its role in diabetes.

## 5. Strengths and Limitations

This current study based on bioinformatics revealed some interesting findings on the relation between T2D and OSCC. It remains, however, limited on the absence of a validation in clinical or laboratory setting. The findings are only a basis for future research in the field and identified several biomarker candidates. The following limitations are of particular relevance and need to be considered in this respect. No validation was performed for the analysis, and therefore, the results are restricted to a hypothetic level. Moreover, there is insufficient information on the sample, especially the patient-related information like age, gender, smoking, and comorbidities. Those parameters might have influenced the current study's findings. Thereby, sample size is limited on the available data and the sample has a potential bias. Overall, the transferability and clinical consequences of the findings remain limited and speculative. Therefore, subsequent studies are required to validate the results. First, findings should be used as a basis for an in vitro or animal model and then transferred to clinical context.

## 6. Conclusion

A genetic cross-talk between T2D and OSCC points out on the close relationship between those two diseases. Plasmacytoid dendritic cell- and activated dendritic cell-related genes were associated with the survival of T2D-related OSCC. Four genes, i.e., ABCD1, C1QC, CSE1L, and PSMC4, were the most important immune-related cross-talk genes between T2D and OSCC.

## Figures and Tables

**Table 1:** Datasets of TCGA-OSCC and T2D.

	TCGA-OSCC	T2D		
Datasets	TCGA-OSCC	GSE184050	GSE23561	GSE26168
Platform		GPL11154	GPL10775	GPL6883
Experimental type	High-throughput sequencing	High-throughput sequencing	Array	Array
Case	305	50	8	9
Control	44	66	9	8
Total	349	116	17	17

**Table 2:** DEGs of OSCC and T2D.

	OSCC	T2D(GSE184050)
*p* value	*p* < 0.01	*p* < 0.05
|Log2(FC)|	|Log2(FC)| > 0	
DEG up	2696	566
DEG down	9182	1672
Total DEG	11878	2238
Cross-talk gene	1008	

## Data Availability

The datasets used and/or analyzed during the current study are available from the corresponding author on reasonable request.
